# Quantification of Cell-Free *mSHOX2* Plasma DNA for Therapy Monitoring in Advanced Stage Non-Small Cell (NSCLC) and Small-Cell Lung Cancer (SCLC) Patients

**DOI:** 10.1371/journal.pone.0118195

**Published:** 2015-02-12

**Authors:** Bernd Schmidt, Julia Beyer, Dimo Dietrich, Ines Bork, Volker Liebenberg, Michael Fleischhacker

**Affiliations:** 1 Clinic for Internal Medicine I, Department of Pneumology, UKH, Halle/Saale, Germany; 2 University Hospital Bonn, Institute of Pathology, Bonn, Germany; 3 Metanomics Health GmbH, Berlin, Germany; H. Lee Moffitt Cancer Center & Research Institute, UNITED STATES

## Abstract

**Purpose:**

Most patients suffering from advanced lung cancer die within a few months. To exploit new therapy regimens we need better methods for the assessment of a therapy response.

**Material and Methods:**

In a pilot study we prospectively enrolled 36 patients with advanced NSCLC and SCLC (34 stage IV, 2 stage IIIB) of whom 34 received standard platinum-based chemo/radiotherapy and two were treated with a tyrosine kinase inhibitor. We measured the levels of extracellular methylated *SHOX2* DNA (*mSHOX2*) in plasma before and during therapy until re-staging. The *mSHOX2* analysis was blinded with respect to the clinical data making it an observational study.

**Results:**

According to the re-staging of 31 first-line patients, 19 patients were classified as non-responders while 12 patients were in the responder group. We observed a tight correlation between radiological data and the change of plasma *mSHOX2* level as the equivalent for a therapy response. A ROC analysis showed a high discriminatory power for both patient groups already one week after therapy start (AUC 0.844). Additionally, a Kaplan-Meier and Cox Proportional Hazards analyses revealed a strong relationship between survival and plasma *mSHOX2* value p≤0.001 (hazard ratio 11.08) providing some evidence for *mSHOX2* also being a predictive marker.

**Conclusion:**

The longitudinal measurement of extracellular plasma *mSHOX2* DNA yields information about the response to cytotoxic treatment and allows an early assessment of treatment response for lung cancer patients. If confirmed in a larger study this would be a valuable tool for selecting and guiding a cytotoxic treatment.

## Introduction

Lung cancer is still a health problem and in 2012 there were more than 409,000 new lung cancer cases in Europe [1]. The five-year survival rates for lung cancer at all stages is 16% and only slightly better than it was 30 years ago [2]. In recent years several new therapy regimens were introduced including a variety of different multimodal treatments for patients with locally advanced, late stage and metastatic disease [3]. Advances in the systemic therapies not only lead to an improved survival but also to a reduction of cancer-related symptoms and a higher quality of life [4]. Nevertheless, the therapeutic window is still small, and it is important to have a method for an early response evaluation to choose the optimal therapy. The method of choice for the assessment of treatment response is a re-staging after two to four cycles of systemic therapy (i.e. after 6 to 12 weeks) using an imaging technique like CT, MRI, or PET. Apart from the high costs, these techniques are not very sensitive [5][6]. An alternative would be the use of biomarkers like *CYFRA-21*, *SCCA*, *CEA* and *CA-125* for NSCLC patients and *ProGRP* and *NSE* for SCLC patients to correlate them with therapy response [7]. Unfortunately there is no universal marker that useful for all different lung cancer histologies and there is not enough evidence for any of them to be routinely used in the clinic.

Mandel and Metais were the first to describe their observation of the presence of extracellular nucleic acids in humans [8]. Tumor-associated genetic alterations can be found in cell-free nucleic acids isolated from all different body fluids [9,10][11]. According to our current knowledge all tumor-associated alterations found in tumor cells can also be detected in extracellular nucleic acids, including epigenetic alterations associated with the development of malignant tumors. DNA methylation and cytosine methylation are a hallmark of mammalian chromatin, play a role in the regulation of development and are important in basic biological processes like embryogenesis and cell differentiation [12] [13]. As such, DNA methylation regulates gene transcription and epigenetic alterations in oncogenes and tumor suppressor genes and are of key importance to cancer development [14]. Recently, the methylation of the *SHOX2* gene (*mSHOX2*) has been described as a novel and powerful marker for an early detection of patients with lung cancer based on the analysis of bronchial aspirates and plasma [15] [16] [17], the evaluation of paramalignant and malignant pleural effusions [18], the examination of needle aspirates for lung cancer staging [19] and as a predictor for outcome in NSCLC patients [20]. This study was performed to evaluate i) whether the quantitative analysis of *mSHOX2* plasma DNA correlates with treatment response in lung cancer patients and ii) to determine the best time for performing the analysis of this biomarker.

## Material and Methods

### Patients

We prospectively enrolled 36 patients which were consecutively referred to our outpatient clinic for diagnosis and treatment of lung cancer. We included patients with a late stage/advanced histologically proven lung tumor (independent of the typ of lung cancer) who were eligible for a chemo/radio-chemotherapy and had signed a written consent to participate in this study. When the clinical data wase combined with the *mSHOX2* measurements we realized that five patients had received a treatment before enrollment in our study. All other 31 patients received a first-line therapy. The details of the clinical data of all patients are summarized in Tables 1–3. The specimens for the histopathological diagnosis were obtained by bronchoscopy and/or computed tomography (CT). All but one patient received a standard platinum-based combination chemotherapy and if necessary an additional radiotherapy according to existing guidelines. [21]. As part of the diagnostic workup all lung cancer patients are screened for EGFR mutations. Patients UKH10 and UKH 031 demonstrated an activating EGFR mutation and were treated with Erlotinib. After three therapy cycles the patients were re-staged by physicians of the local tumor board based on repeat-CT. The response evaluation and the assignment of the patient as responders and non-responders, respectively were carried out according to RECIST v1.1 criteria. The study has been approved by the Institutional Review Board (IRB) at the University Hospital of Halle/Saale. Informed consent (written) was obtained from all donors.

**Table 1:** Clinical data of patients not responding to the therapy.

Patient ID	Smoking status / pack years	Histology	Stage (UICC/AJCC)
UKH 003	never	Adenocarcinoma	IV
UKH 005	ex-smoker / 35	SCLC	IV
UKH 009	ex-smoker / 22	SCLC	IV
UKH 011	yes / 37	NSCLC, large cell	IV
UKH 014	ex-smoker / 35	undifferentiated carcinoma	IV
UKH 016	ex-smoker / 45	undifferentiated carcinoma	IV
UKH 017	ex-smoker / 6	Adenocarcinoma	IV
UKH 018	ex-smoker /50	Squamous cell carcinoma	IIIB
UKH 019	ex-smoker / 60	Adenocarcinoma	IV
UKH 023	ex-smoker / 36	undifferentiated carcinoma	IV
UKH 024	ex-smoker / 30	Adenocarcinoma	IV
UKH 027	ex-smoker / 24	SCLC	IV
UKH 028	ex-smoker / 50	Sqamous cell carcinoma	IV
UKH 033	yes / 23	Sqamous cell carcinoma	IV
UKH 035	ex-smoker / 27	Adenocarcinoma	IV
UKH 036	yes / 30	Adenocarcinoma	IV
UKH 038	yes / 26	Adenocarcinoma	IV
UKH 039	yes / 20	Adenocarcinoma	IV
UKH 041	yes / 20	Adenocarcinoma	IV

Clinical data for first line patients who did not respond to the therapy. Fifteen of the patients were male and four were female. The median age of this patient group is 63 years.

**Table 2:** Clinical data of first-line patients responding to the therapy.

Pat ID	Smoking status / pack years	Histology	Stage (UICC/AJCC)
UKH 007	ex-smoker / 35	SCLC	IV
UKH 012	yes / 42	SCLC	IV
UKH 015	ex-smoker / 17	Adenocarcinoma	IV
UKH 022	ex-smoker / 35	undifferentiated carcinoma	IV
UKH 025	ex-smoker / 25	SCLC	IV
UKH 026	yes / 40	Adenocarcinoma	IIIB
UKH 029	ex-smoker / 29	Adenocarcinoma	IV
UKH 030	yes / 33	Adenocarcinoma	IV
UKH 031	never	Adenocarcinoma	IV
UKH 034	ex-smoker / 15	Adenocarcinoma	IV
UKH 040	never	Adenocarcinoma	IV
UKH 042	ex-smoker / 33	Sqamous cell carcinoma	IV

Ten of the patients were male and two were female. The median age of this patient group is 61.5 years. Patient UKH 031 demonstrated an EGFR mutation and was treated with TKI.

**Table 3:** Clinical data of second-line patients.

Pat Id	Smoking status / pack years	Histology	Stage (UICC/AJCC)	Responder/ non-responder
UKH 001	never	Adenocarcinoma	IV	Responder
UKH 002	yes / 6	Squamous cell carcinoma	IV	Responder
UKH 010	ex-smoker / 30	Adenocarcinoma	IV	non-responder
UKH 020	ex-smoker / 35	Squamous cell carcinoma	IV	non-responder
UKH 037	yes / 34	Adenocarcinoma	IV	non-responder

In this group were four male and one female patients. The only female patient in this group (UKH 001) did respond to therapy. The median age of this patient group is 64 years. Patient UKH 010 demonstrated an EGFR mutation and was treated with TKI.

### Preparation of plasma samples

We obtained 2 × 8.5 mL EDTA blood from all patients at the time of diagnosis (pre-therapy = baseline) and every time the patients were checked for their blood counts or when they received a chemotherapy treatment (usually at intervals of 7 to 10 days). The patients were followed until the end of three therapy cycles, i.e. the time of re-staging. The plasma was prepared by spinning the blood samples (within 1 to 2 hrs after blood drawing) for 15 min at 500x g. After careful transfer of the plasma supernatant into a new tube the sample was spun for a second time for 15 min at 2500x g. All samples were stored in 3–4 mL aliquots at -80°C until use.

### Real-time quantification of *mSHOX2* plasma DNA

Free-circulating DNA from 3.5 mL plasma samples was isolated and bisulfite converted using the Epi proColon Plasma Quick Kit (Epigenomics AG, Berlin, Germany). DNA isolation and bisulfite conversion was carried out following the instruction with minor modifications. The DNA was finally eluted from the beads with 68 μL elution buffer. Together with the patient samples we measured a calibrator sample (i.e. 5 ng artificially methylated bisulfite converted DNA). The sensitive and quantitative qPCR analysis of *mSHOX2* was carried out as previously described [16])[18]. Each sample was measured in six PCR replicates and a relative methylation value (= PMR, percent methylation reference) for *mSHOX2* was calculated using the adapted ΔΔCT method [16]. The *mSHOX2* DNA quantification was performed after all prospectively collected plasma samples were complete, i.e. making this analysis an observational study.

### Statistics

Differences of methylation levels (PMR) in blood plasma of reponders and non-responders at base line and follow-up time points 1–8 were tested using unpaired two-sample Wilcox tests (Mann Whitney) given that the PMR data was not normally distributed. The p-values were Bonferroni corrected. Other descriptive data characteristics used were median and median absolute deviation (MAD). Responder Operator Characteristics (ROC) curves were used to visualize the capability of the *SHOX2* marker to discriminate between responders and non-responders at different time points. Overall survival was calculated using the Kaplan-Meier and univariate Cox Proportional Hazards regression models. The analyses were carried out with the SPSS 21 software package (IBM, Armonk, NY) and R, respectively [22].

## Results

**Fig 1:**
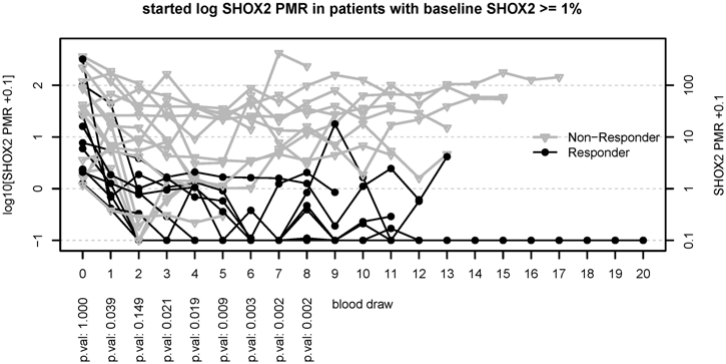
Trend curves for patients responding (black curves) and not responding (gray curves) to the therapy. The patients included in this figure are limited to the ones with a baseline mSHOX2 value of at least 1% PMR. The first blood draw (x = 0) is the point of diagnosis, i.e. before treatment and defines the baseline methylation of SHOX2. For the first eight blood draws Bonferroni corrected p-values from unpaired two sample Wilcox tests are given at the bottom.

The assignment of the 36 prospectively enrolled patients into responders and non-responders, respectively was completely independent of the *mSHOX2* analysis. Thirty one patients received a first-line therapy (Tables 1 and 2) while five patients had been treated before enrollment into the study (Table 3). All but two patients demonstrated a wild-type *EGFR* gene and received a standard platinum-based chemotherapy, while the two patients with an activating *EGFR* mutation were treated with TKI. Seven of these 31 patients demonstrated a baseline PMR value ≤ 1% which is assumed to be the level of technical/biological variance. The clinical data from the 31 patients are summarized in Tables 1 and 2. All patients who clinically responded to the therapy demonstrated a decrease of their *mSHOX2* plasma DNA (Fig. 1). In this group of responders a decrease of *mSHOX2* DNA was seen in most of the patients already at the time of first blood draw (i.e. day 7–10 after therapy start). The median PMR values of the patients responding to the therapy were 4.06% at baseline and dropped to 0.62%, 0.12%, 0.05% at blood draws 1, 2 and 3 after therapy start. In contrast, 8/19 of the non-responding patients demonstrated a reduction of *mSHOX2* after start of therapy but the *mSHOX2* levels did not change as much as in the responding patients (Fig. 1). In fact the median *mSHOX2* values in this group of non-responders were 26.45% at baseline and dropped to 6.86%, 7.78%, 7.96% at blood draws 1, 2 and 3 after therapy start. None of the non-responding patients who demonstrated a *mSHOX2* value of ≥ 1% pre-therapy (ranging from 1.1% to 362%) showed a sustained reduction below 1% PMR. (Fig. 1).This observation holds true also for patient UKH 010 who demonstrated an activating EGFR mutation but did not respond to the TKI therapy (Table 3).

The PMR values are calculated as relative amounts of the *mSHOX2* gene compared to the ß-actin reference gene (*ACTB*). The *SHOX2* locus is frequently amplified in lung tumors (Schneider et al BMC Cancer 2011) and this copy number variation leads to *mSHOX2* PMRs over 100% in patients with very high levels of free circulating tumor DNA.

**Fig 2:**
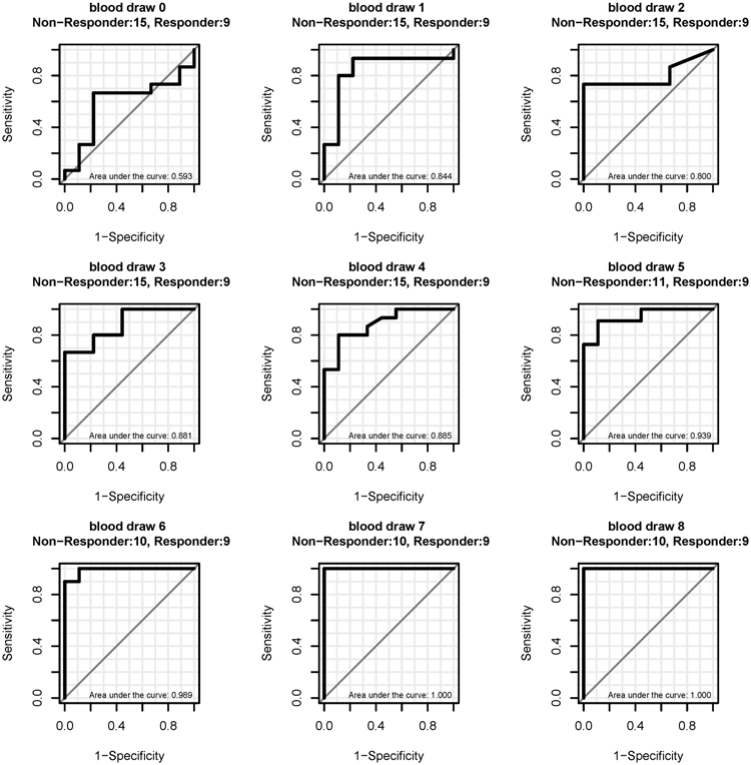
ROC curves for the discrimination of responders from non-responder at different blood draws. Only patients with a baseline PMR ≥ 1% were included. The first blood draw (time 0) is the point before treatment (= baseline methylation). Blood draws 1 to 8 were taken during the therapy at intervals of 7 to 10 days.

The median PMR for *mSHOX2* at baseline was 4.06% for the 12 responders and 26.45% for the 19 non-responders but this difference is not significant. Interestingly, the ROC curve analyses for the 24 patients with a PMR ≥ 1% showed that the baseline values *mSHOX2* did not discriminate between responders and non-responders (area under the curve 0.593), while a discrimination of these two patient populations based on values obtained after start of the therapy demonstrated a high sensitivity and specificity already starting at blood draw one with an AUC of 0.844 which increased to 1.000 at blood draw 7 (Fig. 2). The classification of the patients as responders and non-responders is based on CT scans as the gold standard which was performed by the local tumor board and was completely independent from the *mSHOX2* measurements.

**Fig 3:**
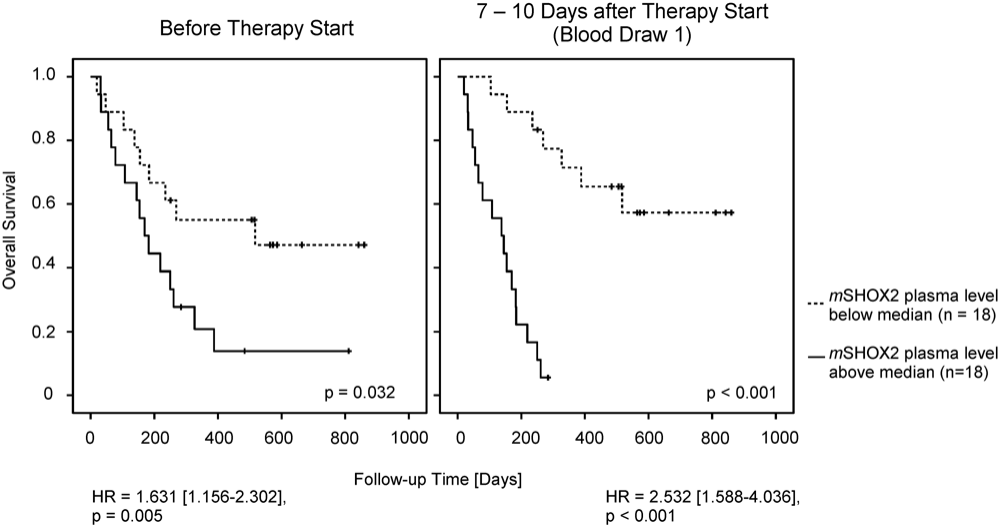
Cox Proportional Hazards and Kaplan-Meier survival analysis of all 36 patients. The median plasma *mSHOX2* value at baseline was 2.88% PMR, while the median one week after therapy start was 2.16% PMR.

A Kaplan-Meier analysis including the 31 patients (i.e. 24 patients with a PMR ≥ 1% plus 7 patients with a PMR ≤ 1%) demonstrated a strong relationship between the survival time and a CT-based assignment of patients into responder and non-responder. The hazard ratio in this calculation is 18 and the P-value ≤ 0.00003 (data not shown). Interestingly, there is a similarly strong relationship between the survival time and the PMR of *mSHOX2*. When the PMR values from all 36 patients were used for a Kaplan-Meier analysis we could demonstrate that even the plasma *mSHOX2* baseline levels showed a trend toward significance (Fig. 3). At blood draw one this difference reached statistical significance which might be interpreted as evidence that this marker also has a predictive value.

## Discussion

Due to more powerful drugs [23] and the introduction of a targeted therapy for molecularly selected patient subgroups like patients with an *EGFR* activation mutation, impressive improvements in the treatment of lung cancer patients were achieved [24]. Additionally, more effective maintenance regimens have shown beneficial effects for patients with advanced stage NSCLC. There is also a survival advantage for patients who are treated with second-line chemotherapy as compared to best supportive care and clinical trials testing new combinations in the second line setting for refractory disease were initiated [25][26]. In order to select the best treatment options, a rapid, specific and sensitive method for the assessment of a therapy response is of crucial importance. The standard procedure for advanced stage lung cancer patients after induction therapy is a CT scan to evaluate the tumor response [21]. Apart from the costs, the sensitivity of this imaging technique is not very high and the inter-observer variability in the measurement of the tumor size is prone to misinterpretation of tumor response [27].

In the last few years several biomarkers have been tested for their usefulness as an indicator for therapy monitoring. Amongst them were eight immunohistochemical biomarkers, none of which could predict chemotherapy response and survival rate and there was only a weak correlation between marker level and treatment response [28]. Neuron specific enolase (*NSE*) was used for monitoring SCLC patients and found to be only useful in patients with an increased pre-treatment level [29]. The levels of lactate dehydrogenase and chromogranin A did not correlate with treatment response [30]. A longitudinal measurement of soluble interleukin 2 receptor demonstrated a reduction in serum concentration during a therapy but was not a sign for disease remission [31] and thymidine kinase was unable to discriminate between the various response groups of lung cancer patients [32]. There are some additional biomarkers for therapy monitoring in lung cancer patients like *CYFRA 21–1* and nucleosome levels which might be better suited but none of them is routinely used in the clinic [33] [34] [35] [36]. In addition there is a growing number of genes which are shown to be hyper- or hypomethylated in lung cancer patients which might be useful as biomarkers [37]. The most frequently analyzed genes like *p16*, *DAPK*, *APC*, *RASSF1A*, *MGMT*, *FHIT*, *RARß and GSTP1* were applied as a means of detecting lung cancer or as prognostic factor but none of them had been used for therapy monitoring. Interstingly, all of these methylation markers could not only be detected in tissue but were also found in extracellular nucleic acids isolated from bronchial lavage supernatants, sputum, plasma or serum [37]. The aim of this analysis was to answer the question whether a quantitative measurement of *mSHOX2* plasma DNA using a real-time PCR is a useful tool to follow advanced stage lung cancer patients receiving chemo/radio-chemotherapy. Therefore we enrolled all eligible patients who were consecutively admitted to our outpatient department. We considered this approach a proof-of-principle study and for this reason we were more interested in the inclusion of as many patients as possible rather than the establishment of a uniform patient cohort. As a consequence the histologic distribution of the patients included in this analysis does not match exactly the figures given in the literature [38].

Our results demonstrate that a quantitative determination of plasma *mSHOX2* DNA appears to be useful for the monitoring of a treatment response for advanced stage lung cancer patients. The turn-over rate of cell-free DNA is rather high as the half-life of extracellular nucleic acids was determined to be less than six hours in an animal model [39]. This correlates well with our observation of a fast and strong decline of plasma *mSHOX2* DNA in patients responding to the therapy which holds true for the monitoring of NSCLC and SCLC patients alike. An additional advantage of this method is its applicability for patients with a very low pre-therapeutic *mSHOX2* value. We demonstrated that a *mSHOX2* measurement taken one week after the start of a therapy is able to divide between responders and non-responders with a very high specificity. If this result can be verified in a large study, physicians have the possibility to switch therapies or spare patients from an invalid therapy. Additionally, we used the data from all 36 patients, i.e. including the patients with a *mSHOX2* baseline value of zero and the second-line patients for a Kaplan-Meier survival curve analysis. Interestingly, we demonstrated that the patients with a baseline plasma *mSHOX2* level below the median had a slightly longer survival time. When this analysis was performed at blood draw one after therapy start this difference between patients responding to the therapy and non-responders was statistically significant with a p ≤ 0.001. The question whether patients with a very low *mSHOX2* baseline value behave differently from patients with a high(er) *mSHOX2* level and whether it might be possible to define a pre-therapeutic cut-off value which has a predictive meaning has to be answered in a large study which will be conducted in a multicentric approach.

From our results we conclude that the measurements of the plasma *mSHOX2* level are a reflection of the clinical course of late stage lung cancer patients receiving a systemic treatment. Such an analysis allows a more rapid and sensitive determination of tumor response and therapy monitoring than a CT scan. Whether the measurement of extracellular *mSHOX2* DNA in plasma might also have a predictive value needs to be demonstrated in future trials.
